# Deficiency of apoptosis‐stimulating protein two of p53 ameliorates acute kidney injury induced by ischemia reperfusion in mice through upregulation of autophagy

**DOI:** 10.1111/jcmm.14094

**Published:** 2019-01-23

**Authors:** Jing Ji, Xiaoshuang Zhou, Ping Xu, Yafeng Li, Honglin Shi, Dexi Chen, Rongshan Li, Hongbo Shi

**Affiliations:** ^1^ Beijing Youan Hospital Capital Medical University Beijing China; ^2^ Beijing Institute of Hepatology Capital Medical University Beijing China; ^3^ Shanxi Provincial People’s Hospital Affiliated to Shanxi Medical University Taiyuan China

**Keywords:** acute kidney injury, apoptosis‐stimulating protein two of p53, autophagy

## Abstract

Acute kidney injury (AKI) has become a common disorder with a high risk of morbidity and mortality, which remains major medical problem without reliable and effective therapeutic intervention. Apoptosis‐stimulating protein two of p53 (ASPP2) is a proapoptotic member that belongs to p53 binding protein family, which plays a key role in regulating apoptosis and cell growth. However, the role of ASPP2 in AKI has not been reported. To explore the role of ASPP2 in the progression of AKI, we prepared an AKI mouse model induced by ischaemia reperfusion (I/R) in wild‐type (ASPP2^+/+^) mice and ASPP2 haploinsufficient (ASPP2^+/−^) mice. The expression profile of ASPP2 were examined in wild‐type mice. The renal injury, inflammation response, cellular apoptosis and autophagic pathway was assessed in ASPP2^+/+^ and ASPP2^+/−^ mice. The renal injury, inflammation response and cellular apoptosis was analysed in ASPP2^+/+^ and ASPP2^+/−^ mice treated with 3‐methyladenine or vehicle. The expression profile of ASPP2 showed an increase at the early stage while a decrease at the late stage during renal injury. Compared with ASPP2^+/+^ mice, ASPP2 deficiency protected mice against renal injury induced by I/R, which mainly exhibited in slighter histologic changes, lower levels of blood urea nitrogen and serum creatinine, and less apoptosis as well as inflammatory response. Furthermore, ASPP2 deficiency enhanced autophagic activity reflecting in the light chain 3‐II conversion and p62 degradation, while the inhibition of autophagy reversed the protective effect of ASPP2 deficiency on AKI. These data suggest that downregulation of ASPP2 can ameliorate AKI induced by I/R through activating autophagy, which may provide a novel therapeutic strage for AKI.

## 1 INTRODUCTION

Acute kidney injury (AKI) is now well known as a worldwide public health concern with high morbidity, mortality and healthcare costs. According to statistics, about 13.3 million people suffer from AKI per year and about 1.7 million patients dead of AKI per year, most of whom come from developing countries.^1^, ^2^, ^3^ AKI often develops into chronic or end‐stage renal disease, but reliable and effective therapeutic intervention has been not developed to improve survival, limit damage or accelerate recovery except dialysis.^4^, ^5^ Therefore, the study of damage mechanism is important for AKI.

Apoptosis‐stimulating protein two of p53 (ASPP2), also known as 53BP2L, is a proapoptotic regulator of p53‐binding protein family.^6^ It was first identified as a tumor suppressor and an activator of p53 family members,^7^, ^8^ and play a critical role in regulating apoptosis and cell growth.^9^ A few studies on ASPP2 in kidney indicated that ASPP2 may play an important role in the renal desease.^10^ Li et al found that decreased ASPP2 was correlated with high grades and poor outcomes of renal cell carcinoma, which related to that ASPP2 downregulation promoted epithelial to mesenchymal transition and increased resistance to 5‐Fluorouracil (5‐FU)‐induced apoptosis.^11^ Wang et al uncovered that ASPP2 contributed to renal mesenchymal to epithelial transition in C57BL/6 mice through bingding Par3 amino‐terminus, which suggested that ASPP2 may inhibite renal carcinoma metastasis.^12^ However, whether ASPP2 is involved in the process of AKI or plays certain roles remains unclear.

Autophagy is an evolutionarily conserved multi‐step process depend on intracellular lysosome catabolism, which is a protective mechanism for maintaining cellular homeostasis and cell survival under pathological stress conditions.^13^, ^14^ In 1963, Christian de Duve coined the term “autophagy” to describe this phenomenon.^15^ Autophagy in renal cell can be activated under stress states including cell starvation, ischemia and hypoxia, nutrient and growth‐factor deprivation, oxidant injury and certain genetic toxicants, which were also involved in the procedure of AKI.^16^, ^17^, ^18^, ^19^ Xie et al observed that ischemia preconditioning can attenuate renal ischemia reperfusion (I/R) injury by activating autophagy.^20^ Melk et al found that the upregulation of autophagy is a prosurvival mechanism of acute tubular cell injury in ischemia AKI in Atg5 knockout mice. Interestingly, 30 days after I/R, the mice presented less renal tubular injury and low autophagic level, which suggested a long‐term benefit of autophagic inactivation.^21^ Hence, early autophagic activation plays a protective role in AKI, but a long‐period or persistent autophagic activation may exacerbate cell damage.^21^, ^22^

Studies have shown that ASPP2 can regulate cellular autophagy. A recent study found that ASPP2 negatively regulated basal autophagy with the reduced expression of light chain 3 (LC3)‐II and other autophagy‐related genes in pancreatic cancer cell lines by decreasing AMPK and TSC2 phosphorylation in the AMPK‐mTOR pathway.^23^ Wang et al discovered that ASPP2 suppressed RAS‐induced autophagy via competing to bind Atg5‐Atg12 complex with Atg16L1.^24^ Liu et al found that ASPP2 can facilitate autophagic apoptosis by releasing Beclin‐1 from cytoplasmic Bcl‐2‐Beclin‐1 complexes.^22^ All these findings indicate that ASPP2 is likely to an indispensable regulator in autophagy.

Based on the information all above, we hypothesized that ASPP2 played a vital role in the progression of AKI through autophagic pathways. To confirm this speculation, we used a classical renal I/R model to study the effect of ASPP2 on AKI and its regulatory mechanism.

## 2 MATERIALS AND METHODS

### 2.1 Animals

Male ASPP2^+/+^ Balb/c mice (aged 6‐8 weeks, weighed 25‐30 g) were originally purchased from the Animal Center of the Academy of Military Medical Sciences (Beijing, China). Male ASPP2^+/−^ Balb/c mice (aged 6‐8 weeks, weighed 25‐30 g) were provided by the Beijing Institute of Hepatology. All animals were housed in a specific pathogen‐free facility and maintained in a 12‐hour dark‐light cycle at 22‐24°C and 30%‐40% humidity. All of the mice were active normally and had glossy hair before the start of experiments. Human care was provided to all animals in accordance with the guidelines of the Capital Medical University Animal Care Committee.

### 2.2 **Renal **I/R** experiments**

Age/sex‐matched (6‐8 weeks) male mice were used in each genetic group. Wild‐type (ASPP2^+/+^) and ASPP2 haploinsufficient (ASPP2^+/−^) mice were subjected to renal I/R surgery as described.^25^ Briefly, mice were anaesthetized and were kept on a homeothermic pad to maintain body temperature at 36.5°C. Front incisions were made to expose both renal pedicles for bilateral clamping with nontraumatic microsurgical vascular clips to induce 45 minutes of renal ischaemia. The clamps were released for reperfusion at the indicated times and then the surgical wounds were closed. Control animals were subjected to sham operation by anaesthesia and laparotomy only without renal pedicle clamping. After indicated durations of reperfusion (12, 24, 48, 72 hours), blood and kidney tissues were collected for the following examination. To examine the effect of autophagy inhibition, 3‐methyladenine (3‐MA, 1 mg/kg; Sigma) was injected into mice via tail vein 2 hours before I/R All animal procedures were conducted according to a protocol approved by the Ethics Committee of Beijing Youan Hospital.

### 2.3 Renal function detection

The renal function was assessed by measuring blood urea nitrogen (BUN) and serum creatinine (Scr) levels. Blood samples were collected and then centrifuged at room temperature. The serum samples were analysed by the Clinical Laboratory Center of Capital Medical University.

### 2.4 Histological analysis

Kidney tissues were fixed with 4% paraformaldehyde and embedded in paraffin. The tissues were then sectioned at 4 μm and renal injury was evaluated histologically with light microscopy by hematoxylin‐eosin (HE) staining. Morphological assessments were performed by an experienced renal pathologist who did not know the groups. More than 10 fields including both cortex and outer medulla were randomly selected, and injury was scored according to the percentage of damaged tubules as outlined by Jablonski et al^26^ Tubular injuries include loss of brush border, tubular dilation, tubular cell necrosis, accumulation of cell debris, cast formation and cell lysis. Scores range from 0 to 4 and higher scores indicate more severe damage: 0 means no damage; 1 means less than 5% damage; 2 means 25%‐50% damage; 3 means 50%‐75% damage; and 4 means more than 75% damage. All images were acquired by an upright light microscope (Leica Microsystems, Mannheim, Germany).

### 2.5 Apoptosis detection

Kidney cortex tissues were frozen in optimum cutting temperature compound and sectioned at 5 μm by freezing microtome (Leica Kryostat, Germany). According to the manufacturer’s instructions, apoptotic cells in kidney tissues were detected by terminal deoxynucleotidyl transferase‐mediated deoxyuridine triphosphate nick end labeling (TUNEL) assay, using an in situ apoptosis detection kit (NanJing KeyGen Biotech, Jiangsu, China), and nuclei were stained using DAPI (5 μg/mL; Sigma‐Aldrich, St. Louis, MO, USA). For relative quantification, 10 different regions per section and 5 sections per experimental group were selected and the amount of TUNEL‐positive cells of per 100 cells was evaluated. All images were acquired by an inverted fluorescence microscope (Nikon Eclipse E800, Tokyo, Japan).

### 2.6 Western blotting analysis

Frozen kidney cortex tissues were lysed by RIPA Lysis Buffer (Solarbio, Beijing, China) with a protease inhibitor cocktail (Sigma‐Aldrich; 1%). The protein concentration was measured using the bicinchoninic acid protein quantification assay kit (Biomed, Beijing, China). Equal amounts of protein were separated on 12% SDS polyacrylamide gel electrophoresis gels and transferred onto polyvinylidene fluoride membrane (Bio‐Rad, CA, USA). After blocking with 5% nonfat milk in Tris‐hydrochloride buffer containing 0.05% Tween‐20 (TBST), the membranes were then incubated overnight at 4°C with primary antibodies against ASPP2, LC3B (1:1000; Sigma‐Aldrich), SQSTM1/p62, Atg5, Atg7, Beclin‐1, and β‐actin (1:1000; Cell Signalling, CA, USA). After three times washes with Tris‐hydrochloride buffer containing 0.05% Tween‐20 (TBST) for 15 minutes, the membranes were blotted with horseradish peroxidase‐conjugated goat anti‐rabbit or goat anti‐mouse IgG antibody (1:2000; Sigma‐Aldrich) at room temperature for 1 hour. The protein bands were visualized using ECL chemiluminescence reagent (Thermo Fisher Scientific, Rockford, IL, USA) according to the manufacturer’s instructions. Densitometry analysis of the bands were performed using ImageJ software, and the relative levels of protein in each group were normalized to the loading control.

### 2.7 Real‐time reverse transcriptase polymerase chain reaction

Total RNA was extracted from mouse kidney cortex tissue using TRIzol (Thermo Fisher Scientific) according to the manufacturer’s instructions. 1 μg of total RNA were reverse transcribed into cDNA using the mRNA reverse transcription kit (Takara Bio, Tokyo, Japan). The ASPP2, IL‐1β, IL‐6, TNF‐α and GAPDH mRNA levels were detected using SYBR Green PCR Kit (Invitrogen, NY, USA) and a real‐time PCR system (ABI PRISM 7300, MA, USA). The primers used in RT‐RCR as followed as Table 1. Quantification was performed using ΔCt values and relative gene expression was determined by normalizing to GAPDH.

**Table 1:** Sequence of primers for real‐time PCR

Gene	Forward primer (5’‐3’)	Reverse primer (5’‐3’)
ASPP2	CAAGCCTGTGATAGCTGCTG	GGCTTCTAAGTCAGCATCGC
IL‐1β	ACT CCT TAG TCC TCG GCC A	TGG TTT CTT GTG ACC CTG AGC
IL‐6	CTA TAC CAC TTC ACA AGT CGG AGG	TGC ACA ACT CTT TTC TCA TTT CC
TNF‐α	TCT CTT CAA GGG ACA AGG CTG	ATA GCA AAT CGG CTG ACG GT
GAPDH	CGT CCC GTA GAC AAA ATG GT	GAA TTT GCC GTG AGT GGA GT

### 2.8 Statistical analysis

All data are presented as mean ± SD. Statistical significance among multiple experimental values was evaluated using one‐way ANOVA, followed by the post hoc LSD test. The difference between two experimental groups was assessed by an independent sample *t* test. *P* values <0.05 was considered statistically significant. All statistical analyses were conducted using the GraphPad Prism 7.0 software.

## 3 RESULTS

### 3.1 **The expression profile of ASPP2 during renal **I/R** injury in mice**

**Figure 1:**
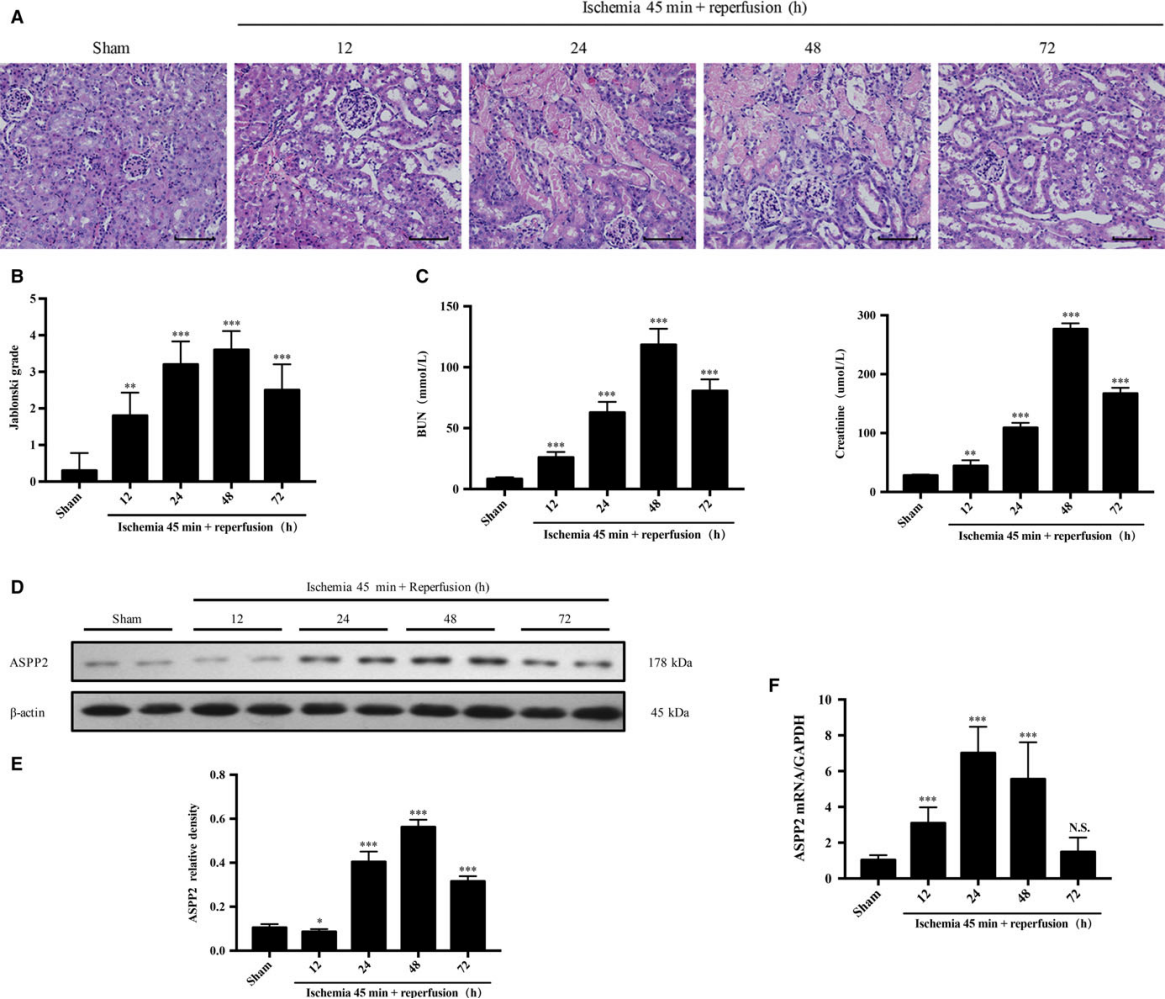
ASPP2 expression profile during AKI induced by I/R Wild‐type mice received a renal I/R surgery of renal pedicles for bilateral clamping. Control mice were subjected to a sham operation only. A, B, Representative H&E staining and Jablonski grade in kidney of ASPP2^+/+^ mice in the sham group and 12, 24, 48, 72 h after renal I/R experiments. Scale bar: 100 μm. C, Serum BUN and Scr levels in ASPP2^+/+^ mice in the sham group and 12, 24, 48, 72 h after renal I/R experiments. D, E, Representative western blotting analysis of ASPP2 in kidney of ASPP2^+/+^ mice in the sham group and 12, 24, 48, 72 h after renal I/R experiments. Quantifications were normalized to β‐actin and showed as relative density. F, The mRNA expression of ASPP2 by quantitive real time PCR in kidney of ASPP2^+/+^ mice in the sham group and 12, 24, 48, 72 h after renal I/R experiments. These experiments were repeated at least three times (n = 6 for each group). Data are presented as mean ± SD. **P < 0.01; ***P < 0.001. Abbreviations: h, hour; NS, no significant difference.

To investigate the role of ASPP2 in AKI, we firstly examined the expression profile of ASPP2 in the renal I/R injury in wild type (ASPP2^+/+^) mice. Data showed that ASPP2 expression displayed a significantly increase at 24 hours, whereas decrease at 72 hours after reperfusion (Figure 1D‐F). Consistently, biochemical markers of renal injury, the BUN and Scr levels showed the same trend as the ASPP2 expression, which has significantly difference between the model group and the sham group (Figure 1C). In addition, histopathology showed that renal injury mainly occurred in the proximal tubules including tubular cells swelling, loss of brush borders, tubular cells coagulation necrosis, tubular dilation and cell lysis, while glomerular lesions were not obvious. The degree of renal injury was scored by Jablonski grade, which indicated that significantly higher damage in model group than that in sham group (Figure 1A,B). ASPP2 expression was positively correlated with the extent of renal injury, which indicated that ASPP2 may play an important role in AKI.

### 3.2 Deficiency of ASPP2 protects mice against renal ischemia‐reperfusion injury

**Figure 2:**
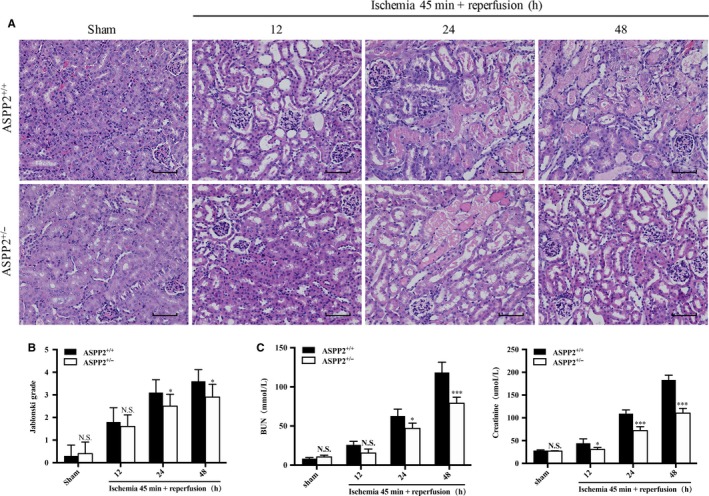
Downregulation of ASPP2 protects mice against renal I/R injury. Mice were treated with renal I/R surgery as mentioned above. Mice were sacrificed at 12, 24, and 48 h after renal I/R experiments, and control mice were subjected to a sham operation only. A, B, Representative H&E staining and Jablonski grade in kidney of ASPP2^+/+^ and ASPP2^+/−^ mice in the sham group and after renal I/R experiments. Scale bar: 100 μm. C, Serum BUN and Scr levels in kidney of ASPP2^+/+^ and ASPP2^+/−^ mice in the sham group and after renal I/R experiments. These experiments were repeated at least three times (n = 6 for each group). Data are presented as mean ± SD. *P < 0.05; ***P < 0.001. Abbreviations: h, hour; NS, no significant difference.

To dissect whether ASPP2 plays a role in the process of renal I/R injury, we established the same renal injury model in both ASPP2^+/+^ and ASPP2^+/−^ mice. We observed that renal histopathology appeared lower Jablonski grade with less loss of brush borders, tubular dilation and cast formation in ASPP2^+/−^ mice than that in ASPP2^+/+^ mice (Figure 2A,B). Besides, the BUN and Scr levels were also lower in ASPP2^+/−^ mice than that in ASPP2^+/+^ mice, which had a statistically significant difference between the two groups (Figure 2C). Taken together, these results revealed that down‐regulation of ASPP2 could alleviate AKI induced by I/R in mice.

### 3.3 **Deficiency of ASPP2 suppresses renal cell inflammation and apoptosis during renal **I/R** injury in mice**

**Figure 3:**
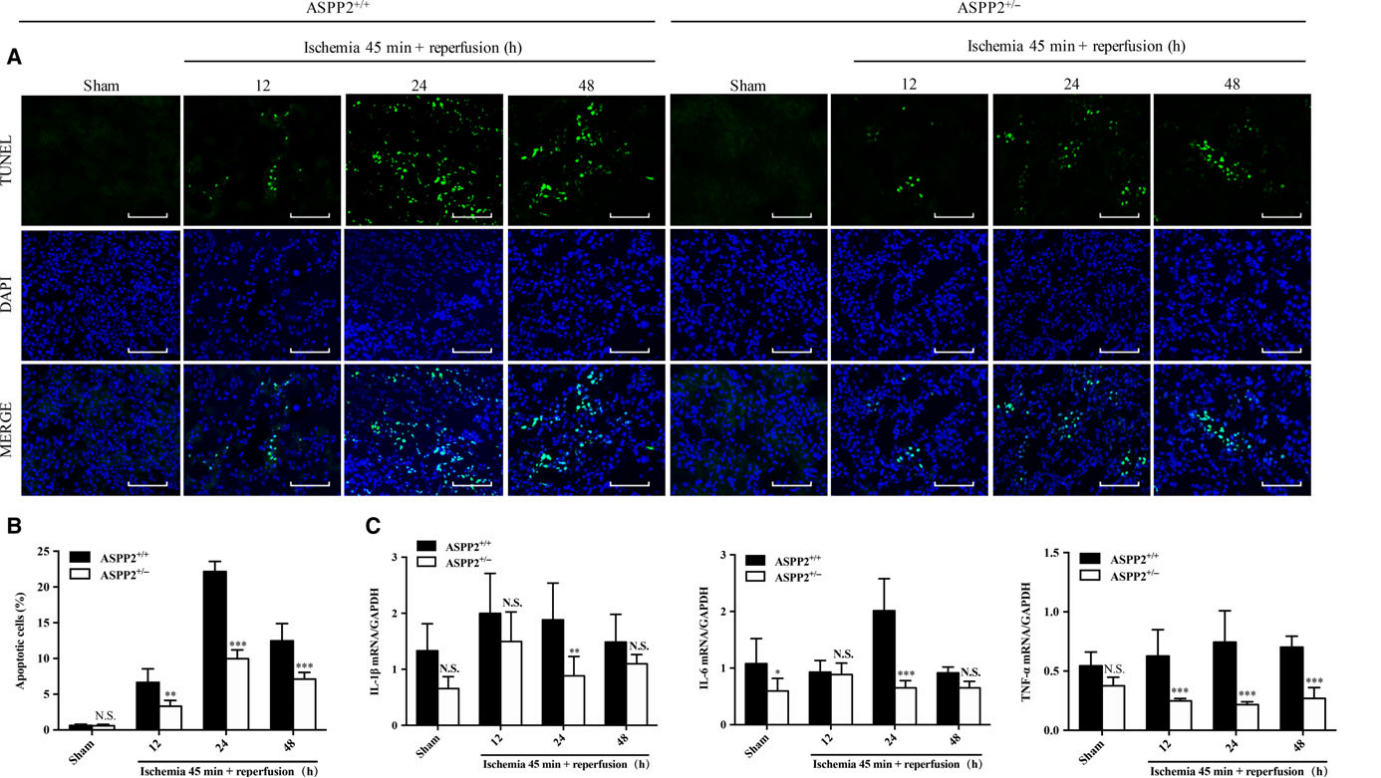
Downregulation of ASPP2 suppresses inflammation and apoptosis in mice with renal I/R injury. Mice were treated with renal I/R surgery as mentioned above. Mice were sacrificed at 12, 24, and 48 h after renal I/R experiments, and control mice were subjected to a sham operation only. A, B, Representative TUNEL images in kidney of ASPP2^+/+^ and ASPP2^+/−^ mice in the sham group and after renal I/R experiments. The TUNEL‐positive cells were counted in 10 microscopic vision fields per section. Five sections per mouse were examined. Scale bar: 100 μm. C, The mRNA expression of inflammation cytokines IL‐6, IL‐1β and TNF‐α by quantitive real time PCR in kidney of ASPP2^+/+^ and ASPP2^+/−^ mice in the sham group and after renal I/R experiments. These experiments were repeated at least three times (n = 6 for each group). Data are presented as mean ± SD. *P < 0.05; **P < 0.01; ***P < 0.001. Abbreviations: h, hour; NS, no significant difference.

Inflammation plays a pivotal role in the pathophysiology of AKI.^27^ To determine the protective mechanism of ASPP2 in renal injury, we then examined inflammatory cytokine expression and cellular apoptosis in kindney tissure of mice. We found that the mRNA expression levels of IL‐1β, IL‐6 and TNF‐α were significantly decreased in ASPP2^+/−^ mice compared with ASPP2^+/+^ mice (Figure 3C). Consistently, similar results had shown that the percentage of apoptotic cells was lower in ASPP2^+/−^ mice than that in ASPP2^+/+^ mice, and significant differences were observed at 24 and 48 hours after reperfusion (Figure 3A,B). Collectively, the data suggested that ASPP2 deficiency can suppress inflammatory responses and cellular apoptosis in AKI induced by I/R in mice.

### 3.4 Deficiency of ASPP2 facilitates autophagy during renal ischemia‐reperfusion injury in mice

**Figure 4:**
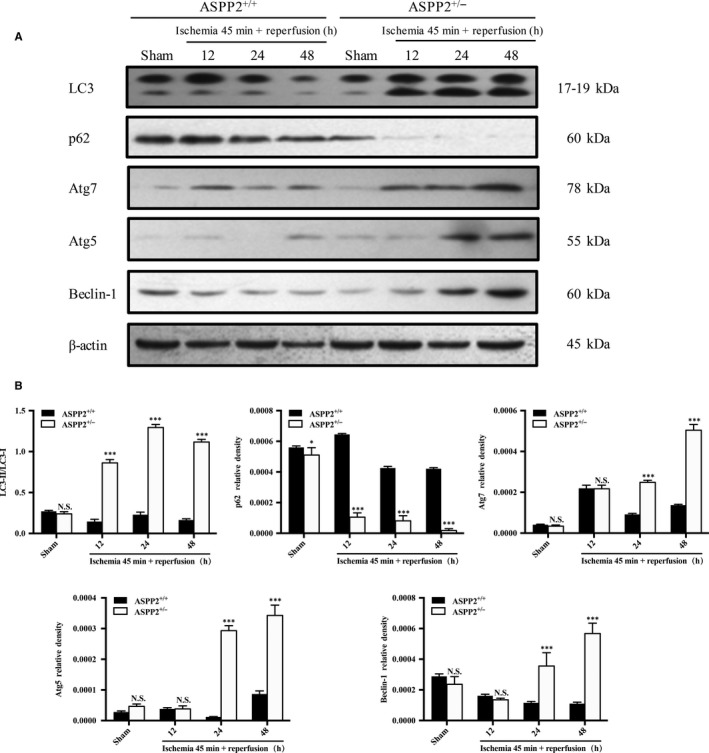
ASPP2 negatively regulates autophagy in mice with renal I/R injury. Mice were treated with renal I/R surgery as mentioned above. Mice were sacrificed at 12, 24, and 48 h after renal I/R experiments, and control mice were subjected to a sham operation only. A, B, Representative western blotting analysis of LC3‐II, p62, Atg5, Atg7 and Beclin‐1 in ASPP2^+/+^ and ASPP2^+/−^ mice in the sham group and after renal I/R experiments. Quantifications were normalized to β‐actin and showed as relative density. These experiments were repeated at least three times (n = 6 for each group). Data are presented as mean ± SD. *P < 0.05; **P < 0.01; ***P < 0.001. Abbreviations: h, hour; NS, no significant difference.

Several studies had reported that ASPP2 may be a key regulator of autophagy.^23^, ^24^ Autophagy could be activated and served as a protective mechanism for cell survival during renal I/R^14^ The conversion of microtubule‐associated protein LC3‐II and the degradation of p62 are widely identified as markers of autophagy flux, in which comversion from LC3‐I to LC3‐II suggests autophagic vacuolization and the amount of LC3‐II is correlated with the extent of autophagosome formation, whereas p62 degradation indicates fusion of autophagosomes with lysosomes.^28^, ^29^ We examined the protein expression of autophagy‐related gene during renal I/R injury in ASPP2^+/+^ and ASPP2^+/−^ mice. LC3‐II conversion and p62 degradation were more significant at 24 and 48 hours after reperfusion in kidney cortex in ASPP2^+/−^ mice than that in ASPP2^+/+^ mice. Furthermore, the time‐dependent reduction of p62 in ASPP2^+/−^ mice compared with ASPP2^+/+^ mice suggested the formation of autophagic flux during renal I/R In addition, the expression of other autophagy‐related genes such as Atg7, Atg5, and Beclin‐1 was also substantially upregulated in ASPP2^+/−^ mice compared with ASPP2^+/+^ mice (Figure 4). Taken together, our data demonstrated that ASPP2 knockdown promoted the activation of autophagy in AKI induced by I/R

### 3.5 **Deficiency of ASPP2 ameliorate renal **I/R** injury through autophagic mechanism**

We confirmed that ASPP2 can negatively regulate autophagic activity in renal injury,^30^ so we finally checked whether the effect of ASPP2 on renal I/R was dependent on autophagy. To verify this point, we used 3‐MA, a class III phosphatidylinositol 3‐kinase inhibitor, to inhibit autophagy.^31^

**Figure 5:**
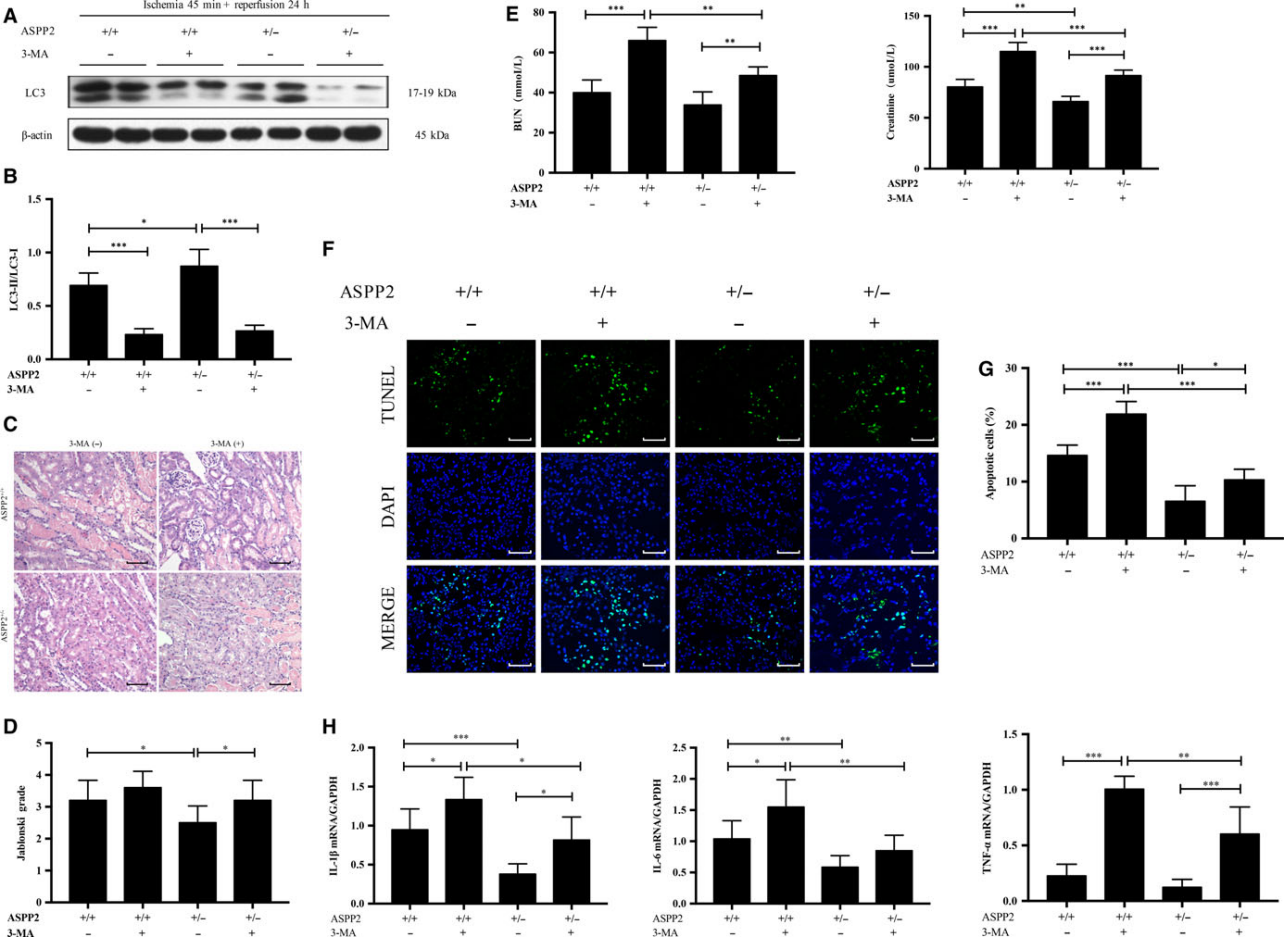
ASPP2 deficiency ameliorates renal I/R damage through autophagic mechanism. Mice were treated with renal I/R surgery as mentioned above. Mice were sacrificed at 24 h after renal I/R experiments. The inhibition of autophagy was performed with a tail vein injection of 3‐MA (1 mg/kg) 2 h before renal I/R experiments. A, B, Representative western blotting analysis of LC3 in ASPP2^+/+^ and ASPP2^+/−^ mice treated with 3‐MA or vehicle. Quantifications were normalized to β‐actin and showed as relative density. C, D, Representative H&E staining of kidney sections in ASPP2^+/+^ and ASPP2^+/−^ mice treated with 3‐MA or vehicle. The degree of damages was showed by Jablonski grade. Scale bar: 100 μm. E, Serum BUN and Scr levels in ASPP2^+/+^ and ASPP2^+/−^ mice treated with 3‐MA or vehicle. F, G, Representative TUNEL images in ASPP2^+/+^ and ASPP2^+/−^ mice treated with 3‐MA or vehicle. The TUNEL‐positive cells were counted in 10 microscopic vision fields per section. Five sections per mouse were examined. Scale bar: 100 μm. H, The mRNA expression of inflammatory cytokines in ASPP2^+/+^ and ASPP2^+/−^ mice treated with 3‐MA or vehicle. These experiments were repeated at least three times (n = 6 for each group). Data are presented as mean ± SD. *P < 0.05; **P < 0.01; ***P < 0.001. Abbreviations: h, hour; NS, no significant difference; 3‐MA, 3‐methyladenine.

3‐methyladenine treatment decreased LC3‐II conversion in mice, suggesting that the inhibition of autophagy by 3‐MA was successful (Figure 5A,B). As we did before, ASPP2 deficiency ameliorated the extent of renal injury, manifesting in low level of serum biochemistry, low Jablonski grade of renal histopathology, low expression of inflammatory cytokines and less cellular apoptosis. 3‐MA injection aggravated pathological lesions of kidney tissues, exhibiting in more loss of brush borders, necrotic tubular cells, and tubular cast formation with a significantly higher Jablonski grade (Figure 5C,D). Consistently, mice with 3‐MA treatment displayed higher levels of BUN and Scr than that without 3‐MA treatment (Figure 5E). 3‐MA treatment also increased mRNA expression of inflammatory cytokines and the amount of apoptotic cells in renal tissues compared with untreated group (Figure 5F‐H). In a word, ASPP2 deficiency ameliorated renal injury induced by I/R, but 3‐MA administration reversed the protective effect of ASPP2 deficiency on renal injury. Therefore, deficiency of ASPP2 can protect mice against renal injury, which was dependent on the activation of autophagy.

## 4 DISCUSSION

As we known, the proapoptotic effects of ASPP2 is tightly linked to its tumour‐suppression function in many animal cancer models including renal carcinoma.^32^, ^33^ However, whether ASPP2 participates in renal injury remains to be further explored. Here, we identified that downregulation of ASPP2 protected mice against AKI by enhancing autophagy, which further suppresses inflammatory response and cellular apoptosis in mouse models.

In China, there are 1.4–2.9 million patients with AKI in 2013 and the hospital admission mortality was estimated to 12.4% according to statistics.^34^ Acute tubular necrosis (ATN) is the most frequent cause of AKI, whereas about two‐thirds of ATN is due to renal I/R.^35^ Hence, we used a renal I/R model to explore the process of AKI. Similar to the previous study of Li et al,^25^ pathological and biochemical changes indicated that an AKI mouse model was constructed successfully (Figure 1).

The ASPP family consists of three members: ASPP1, ASPP2 and inhibitory ASPP. All three proteins have structure similarity in their C‐termini, which contains their signature sequence of Ankyrin repeat, SH3 domain and Proline rich region.^9^ The C‐terminus is the best binding point for ASPPs, and the majority of proteins interacts with ASPPs via ankyrin repeats and SH3 domain.^32^ In 1994, Iwabuchi and his colleagues discovered ASPP2 through a yeast two‐hybrid screen.^36^ Studies had shown that ASPP2 mRNA was highly expressed in the kidney compared with the heart and brain.^37^ Besides, a study indicated that the elevated ASPP2 expression was detected in LPS‐induced maternal inflammation mouse model and human neuroinflammatory disease tissues.^38^ In our study, we found that ASPP2 displayed an increase at early stage and a decrease at late stage during the course of renal I/R The renal pathological and biochemical results showed a similar trend as ASPP2 expression, which indicated that ASPP2 may play an important role in AKI (Figure 1).

There are a few studies on ASPP2 in kidney diseases, which focus on the renal cell carcinoma. Li et al found that ASPP2 was decreased in primary renal cell carcinoma compared with nontumoural kidney controls. ASPP2 was inhibited by histone deacetylatlase 1, which acted by preventing the binding between transcription factor (E2F1) and the ASPP2 promoter.^11^ To explore the role of ASPP2 in the progression of AKI, we prepared an AKI mouse model induced by I/R in wild‐type (ASPP2^+/+^) mice and ASPP2 haploinsufficient (ASPP2^+/−^) mice. Compared with ASPP2^+/+^ mice, renal injury was significantly ameliorated in ASPP2^+/−^ mice, manifesting in lower BUN and Scr levels and lighter damage of tubular (Figure 2). In addition, decreased mRNA expression of inflammatory cytokines also demonstrated an alleviation in kidney inflammation in ASPP2^+/−^ mice. Less TUNEL‐positive cells as well as indicated an apoptosis inhibition in ASPP2^+/−^ mice (Figure 3). Therefore, ASPP2 deficiency exerts a renoprotective effect on AKI.

Autophagy is a highly conservative protein degradation pathway responsible for eliminating unnecessary or dysfunctional cellular components.^13^ It acts as a prosurvival mechanism under stress conditions and plays a critical role in regenerating metabolic precursors and removing subcellular debris to maintain intracellular integrity.^39^ Recent studies uncovered that basal autophagy in the kidney was essential for the maintenance of proximal tubule homeostasis.^40^, ^41^ Kimura et al found an accumulation of damaged mitochondria, p62 and ubiquitin‐positive inclusions in the proximal tubule‐specific Atg5 gene knockout mouse model, which aggravated proximal tubular cell apoptosis and renal dysfunction in mice exposed to I/R^42^ Jiang et al reported that inhibition of autophagy by chloroquine and 3‐methyladenine worsened renal ischemia/reperfusion injury, as indicated by renal function, histology, and tubular apoptosis.^14^

There is close correlation between autophay and inflammation. Similar to our study, some data showed that autophagy inhibition can promote inflammatory factors expression including TNF‐α, IL‐6 and IL‐1β, and autophagy induction can suppress the expression of TNF‐α, IL‐6 and IL‐1β.^43^, ^44^ But the molecular mechanisms how autophagy regulates inflammatory factors expression are still unknown. In current study, autophagy can be activated in inflammatory response through PAMPs (viral, bacterial, funal), cytokines, chemokines and reactive oxygen species. Autophagy activation can reduce inflammation by at least two ways: one is the indirect way to clear damaged organelles and invasive microorganisms, another is the direct way to degrade proinflammatory signalling complexes.^45^

In addition, accumulating evidence suggested that the cytoprotective effect of autophagy can be mediated by negative modulation of apoptosis, which presented in autophagy blocking the induction of apoptosis and in turn apoptotic signalling serving to inhibit autophagy.^46^, ^47^ It is reported that autophagy played multiple roles in inflammatory responses, which can protect renal tubular epithelial cells and podocytes against versatile acute or chronic kidney insults by activating inflammasome and apoptosis.^48^ Su et al observed that renal tissue inflammation and cell apoptosis can be alleviated via the induction of autophagy in AKI following cerebral I/R rats.^49^ Similar to previous findings, our results confirmed that the suppression of autophagy can deteriorate renal tubular injury induced by I/R, leading to renal insufficiency. Additionally, autophagy inhibition also promoted mRNA expression of inflammatory cytokines and renal cell apoptosis, suggesting that autophagy participated in the protective effects of renal injury.

Research showed that ASPP2 can regulate autophagic activity. Chen et al discovered that downregulation of ASPP2 facilitated autophagy in hepatocellular carcinoma (HCC) cells and ASPP2 overexpression blocked starvation‐induced autophagy, whereas decreased ASPP2 expression and improved autophagy were related to poor survival rates in patients with HCC.^30^ Song et al revealed that low expression of ASPP2 suppressed the susceptibility of pancreatic cancer cells to gemcitabine (a chemotherapeutic agent) by enhancing autophagy, and the decreasing of ASPP2 also correlated with poor outcome and low survival rates.^23^ Similarly, we found that the expression of autophagy‐related proteins of Atg5, Atg7 and Beclin‐1 are upregulated in ASPP2 haploinsufficient mice compared to wild‐type mice. More importantly, downregulation of ASPP2 increased LC3‐II conversion and p62 degredation, which indicated that ASPP2 can downregulate autophagy in the AKI mouse model induced by I/R (Figure 4).

We finally analysed whether the protective effect of ASPP2 on AKI was dependent on autophagy. As we expected, ASPP2 haploinsufficient mice treated with 3‐MA had higher Jablonski grade in histopathology, higher levels of BUN and Scr, higher expression of inflammatory cytokines and more renal cell apoptosis than that unteated with 3‐MA, indicating that 3‐MA treatment reversed the protective effect of ASPP2 deficiency on renal injury. Collectively, deficiency of ASPP2 can alleviate AKI, while inhibition of autophagy reverses its beneficial effect, indicating that the protective effect of ASPP2 deficiency on AKI was relied on the activation of autophagy (Figure 5).

**Figure 6:**
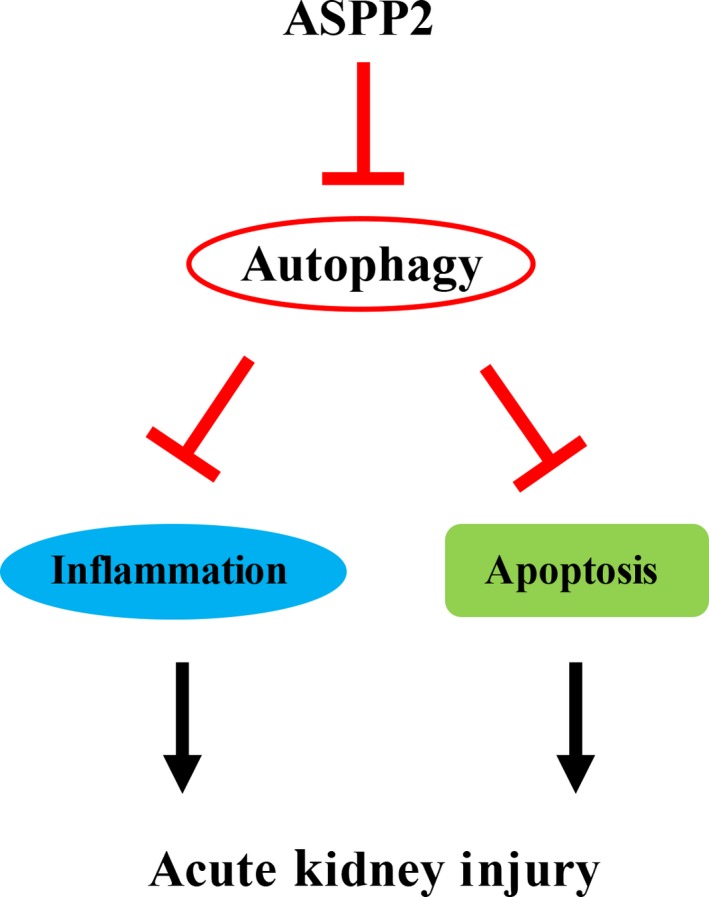
A model of possible role of ASPP2 in regulating acute kidney injury. Our study illustrates that deficiency of ASPP2 can protect mice against renal injury through activation of autophagy which suppresses inflammation and apoptosis. On the contrary, ASPP2 aggravates renal injury through inhibition of autophagy which promotes inflammation and apoptosis

This study revealed that ASPP2 was activated in acute renal injury and acted as a crucial negative regulator of autophagy, which suppressed inflammation responses and cellular apoptosis, consequently resulting in renal injury (Figure 6). Our study identified the critical role of ASPP2 in the development of AKI and provided new sights for lucubrating its underlying biological mechanism. A better understanding of ASPP2 may lead us to new early prevention, diagnosis and even gene therapy approaches in this intractable disease. The combination therapy of ASPP2 and autophagy will be expected to be one of the conceivable treatments for AKI patients in the future.

## CONFLICT OF INTEREST

The authors declare that they have no competing interests.
